# Supplementary material for: Cloud BioLinux: pre-configured and on-demand bioinformatics computing for the genomics community
Source: BMC Bioinformatics. 2012 Mar 19;13:42. doi: 10.1186/1471-2105-13-42 (PMC3372431; doi:10.1186/1471-2105-13-42)
Supplement: Additional file 1 — Supplementary 1 Cloud BioLinux software documentation in the form of a mini, self-contained website. Users need to download and uncompress the .zip file, and open through a web browser the "index.html" file available on the main directory. (ZIP 1823 kb). [file 1471-2105-13-42-S1.ZIP › Cloud-BioLinux-Package-Documentation/docs/samtools.html]

Bio-Linux Software Documentation Pages

Back to search form

## samtools

|  |  |
| --- | --- |
| Name | samtools |
| Description | **Samtools** provides utilities for manipulating alignments in the Sequence Alignment/Map (SAM) format, including sorting, merging, indexing and generating alignments in a per-position format.It also includes an aligment viewer.  The SAM format is a generic alignment format for storing read alignments against reference sequences, supporting short and long reads (up to 128 Mbp) produced by different sequencing platforms. It is flexible, compact and efficient. Alignments from the 1000 Genomes Project are released in SAM format.  The samtools package is prepared by Debian-med and can be installed on Bio-Linux by typing: `sudo apt-get install samtools` There are numerous scripts and programs that come in the samtools package. You can find out more by referring to the man page: after installing samtools, type: `man samtools` or by referring to the remote documentation.  Other tools available on Bio-Linux for handling data from new sequencing technologies are described in the bioinformatics documentation on the NEBC website. |
| Homepage | http://samtools.sourceforge.net/ |
| Remote Documentation | http://www.ncbi.nlm.nih.gov/pubmed/19505943      http://samtools.sourceforge.net/samtools.shtml |
